# Broadwick: a framework for computational epidemiology

**DOI:** 10.1186/s12859-016-0903-2

**Published:** 2016-02-04

**Authors:** Anthony O’Hare, Samantha J. Lycett, Thomas Doherty, Liliana C. M. Salvador, Rowland R. Kao

**Affiliations:** Institute of Biodiversity, Animal Health and Comparative Medicine, University of Glasgow, Glasgow, UK; School of Natural Sciences, University of Stirling, Stirling, UK; The Roslin Institute, University of Edinburgh, Easter Bush, Midlothian. EH25 9RG UK

**Keywords:** Epidemiology, Modelling, Framework, Modularity

## Abstract

**Background:**

Modelling disease outbreaks often involves integrating the wealth of data that are gathered during modern outbreaks into complex mathematical or computational models of transmission. Incorporating these data into simple compartmental epidemiological models is often challenging, requiring the use of more complex but also more efficient computational models. In this paper we introduce a new framework that allows for a more systematic and user-friendly way of building and running epidemiological models that efficiently handles disease data and reduces much of the boilerplate code that usually associated to these models. We introduce the framework by developing an SIR model on a simple network as an example.

**Results:**

We develop Broadwick, a modular, object-oriented epidemiological framework that efficiently handles large epidemiological datasets and provides packages for stochastic simulations, parameter inference using Approximate Bayesian Computation (ABC) and Markov Chain Monte Carlo (MCMC) methods. Each algorithm used is fully customisable with sensible defaults that are easily overridden by custom algorithms as required.

**Conclusion:**

Broadwick is an epidemiological modelling framework developed to increase the productivity of researchers by providing a common framework with which to develop and share complex models. It will appeal to research team leaders as it allows for models to be created prior to a disease outbreak and has the ability to handle large datasets commonly found in epidemiological modelling.

## Background

Mathematical modelling of epidemics has been carried out since the eighteenth century when Daniel Bernoulii used a model to show that life expectancy would be increased when a population was inoculated against smallpox [1]. Since then, mathematical models have increased in sophistication [2–5] and are often accompanied by a wealth of epidemiological and population data [6–10].

It has become easier to collect and store large amounts of data that can be incorporated into epidemiological models, many of which require sophisticated analysis to make efficient use of these data [11–15]. The form of this data is variable but might typically include records of numbers and types of individuals at specific locations at points in time (e.g. a census of cattle on farms), records of movement of individuals between locations, and results of diagnostic tests for disease (symptoms) or specific pathogens. Creating mathematical and computational models and analysing their output requires the development of complex computer code that is usually challenging and disease specific. Therefore a generalised framework upon which complex models can be created, allowing the incorporation of large and flexible datasets whilst maintaining a clear and simple programming model is needed.

Broadwick is a computational framework for developing sophisticated epidemiological models. It consists of several third-party Java libraries and custom packages and removes the boilerplate code and complex data handling tasks so that researchers creating complex models can be more productive in their development. Managers and policy makers can benefit from having a common framework for their projects, facilitating code reuse and sharing amongst research team members and enabling a rapid response rate in the case of a novel disease outbreak.

The components of Broadwick are written in such a way that a user may combine them to rapidly prototype a model for a new specific scenario. Each computational package included in the framework contains sensible default settings that can easily be overwritten. Data can be handled from flat data files or from a database and model parameters can be supplied through a configuration file. The Broadwick code contains a suite of tests that is incorporated into the build system to ensure code validity.

The philosophy driving the development of Broadwick is to have a suite of tools/models written in a common framework that can be available and easily used when a disease outbreak occurs. Having such a common framework allows researchers to quickly develop novel models based on previous ones or to enhance third party models without the investment of time in trying to understand a new code-base.

## Implementation

At its heart, Broadwick is a library that controls how its models are run. It handles its own configuration, data handling and logging (each of which can be configured by the user) allowing developers to concentrate on writing the code to simulate the epidemiology. To make this development process even easier, Broadwick comes with a maven [16] archetype that creates a complete but rather simple runnable model (similar to the ‘Hello World’ example) complete with a useable configuration file, build script, ancillary code and a *NIX shell script for running the project that configures the java runtime environment to be able to run a Broadwick model. Compiling and running this generated project will generate several logging messages as the project is initialised, run and closed. Tutorials of how to create models and run simple examples using the framework, together with examples including an individual based stochastic compartmental model, and models incorporating movements over a network are available from http://epicscotland.github.io/broadwick.html.

**Fig. 1:**
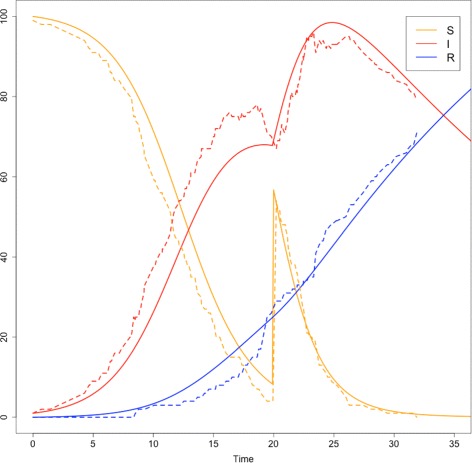
Example of an SIR model using the ODE solver (solid lines) and the stochastic solver (dashed lines) within Broadwick. Both models start with 99 susceptible (S), 1 infected (I) and 0 recovered (R) individuals with *β*=0.004 and *ρ*=0.04 with both solvers adding 50 new individuals at *t*=20 using a *θ*-event

The present version can accommodate two simple models types, event-driven models and ordinary differential equations (ODEs). For event-driven models, a collection of events (reactions) and their transition rates is required by the solver which will determine when an event is fired and which event is to be performed whilst keeping track of the propensities of each agent (though, usually the user will create a AmountManager class to implement this). To use an ODE solver, a collection of equations (the derivatives of some variables) are required by the solver which will apply the 4th order Runge Kutta method to solve the system over time. Future versions of the library will add new ODE solvers (specifically for delayed-ODEs and for variable time-step methods). Examples of running a SIR model using both paradigms are included in the Broadwick distribution which can be modified and built upon to develop more complicated models. Both of these solvers incorporate the observer design pattern [18] to deliver results at the end of each time step (an instance of an observer must be supplied to the solver). These solvers also incorporate a special type of event, which we call *θ*-events that is triggered at specific (user controlled) times. These are useful to, for example, add new susceptible individuals at specific times (to simulate immigration) or to remove infected individuals (to simulate culling events). Figure 1 shows the time series output of a SIR model solved using Broadwick’s stochastic and ODE solvers and incorporating a single *θ*-event to add susceptible individuals.

Broadwick contains several packages for simulating and fitting disease models to observed data (the datasets involved in modelling modern disease outbreaks can be quite large so Broadwick uses an external database to handle this data, see next section for details). The classes Broadwick use for simulating the spread of disease can be combined to quickly create sophisticated models. As an example the Monte Carlo class can run a model (like the ones discussed in the previous paragraph) a specified number of times and generate statistics of the simulation results and the Markov Chain class can generate a sequence of steps in some parameter space.

Markov Chain Monte Carlo can be implemented by adding an acceptor class to the Markov Chain class so successive steps are only accepted if they meet certain criteria (such as minimising a likelihood, or maximising some energy function). Broadwick comes equipped with a Metropolis-Hastings acceptor class which may be used as a template to implement bespoke acceptors (e.g. Metropolis-Hastings algorithm using a log-likelihood or a steepest descent algorithm). Optional classes such as a controller (that specifies conditions for the solver or simulator to stop running) can also be supplied to these simulation classes.

Approximate Bayesian computation (ABC) is an inference techniques that is growing in popularity and one that is supported by Broadwick. A set of priors and a model is supplied to the ABC class which will run the model in the prior space until it converges and reports the calculated posteriors. The ABC class can be used in conjunction with the Markov Chain class to implement the ABC-MCMC allgorithm as proposed by [17]. Controllers and observers can also be supplied to the ABC class as with the Monte Carlo and Markov Chain classes.

This methodology of supplying or overriding functionality within Broadwick is key to the design of the framework. It endeavours to use sensible defaults for each package whilst allowing users to easily overwrite these defaults and combine packages.

### Data

Many epidemiological models require large datasets (e.g. demographic, movement, incidence, location, disease test data) that can represent a prohibitively high cost of computational effort in parsing these datasets before a model can be run. Broadwick overcomes this problem by storing the data in an embedded, in-memory database (once they have been parsed) allowing projects using the framework to use the database thus avoid lengthy start-up times whilst maintaining data integrity and memory efficiency.

Incorporating large datasets (for example individual based movement records) into computational models requires either loading the data into memory at the start of the simulation (which delays the start of the simulation) or configuring a database and interrogating it via complex and often error-prone code (for example JDBC). Broadwick uses a mixed approach by loading all the data into an in-memory database when the code is run for the first time. The database is then saved for future runs, thus avoiding the long start-ups and memory hits associated with data file access. Broadwick uses a SQLite database to store its data, the structure of which is detailed in the Broadwick manual and can also be created and maintained (e.g. periodically updated with new movements and locations) which can then be used by a Broadwick model. This approach may be beneficial if one is performing a long-term study that regularly reports movements or population data that would be required for analysis.

Broadwick accepts comma separated value (CSV) data files for locations, populations, movements and [epidemiological] test data with the layout of each data file described in the projects configuration file.

The location data consists of an identification number (id) of the location (household, animal holding, etc.) and its’ x and y coordinates (there is no requirement on the coordinate system used and the id number can represent either type of location).

The population data can either contain individual level life histories (individual id, date of birth, date of death and location id) or group level information, i.e. the total population size at a location on a specific date (file consists of location id, date and population size). Life history data contain the date and locations of each individuals birth and death.

Test data files consist of a date and the results of a specific test type carried out at a given location or individual (date, test result, location id or individual id) allowing for tests to be recorded at an individual or group level.

Movement data are recorded at different levels depending on whether an individual or groups movement is recorded (following the conventions of the sources of several movement databases, e.g. the Cattle Tracing System (CTS) [19] and ScotEID (for sheep) [20]. Individual movements can be considered either full or directed. Full movements contain the individual’s id, the date of the movement, and the ids of the destination and departure locations. Directed movements contain the id of the individual, the date of the movement, the id of the location and the direction of the movement (‘ON’ or ‘OFF’ specifying if the individual arrived at or left a location, respectively). Group movements, on the other hand, contain the date, number of individuals moved (the batch size), the location id and the movement direction. The tables used in the database are outlined in Table 1.

**Table 1:** Summary of the database schema used in Broadwick

Locations	Contains the id and (x-y or latitude-longitude) coordinates of each location
Populations	Contains the location id and a number of
	individuals at the location with a date.
LifeHistories	Contains the date and locations of each
	individuals birth and death.
Tests	Contains the date and results of a test on a
	given location, group or individual.
FullMovements	Contains an individual’s id and the date
	and id of the departure location and the date
	and id of the destination location.
DirectedMovements	Contains the id, date and direction of a
	movement (‘ON’ or ‘OFF’)
BatchedMovements	Contains the ids, dates of the ‘ON’
	and ‘OFF’ sides of the movement as
	well as the number of individuals moved.

Broadwick contains a facade for interrogating the database through which queries can be run. Several useful methods for interrogating the database are included in this facade e.g. for getting all the movements within a date range (with options to restrict this to movements from or to a specified location), getting all the individuals alive at a given date or all the tests carried out at a specific location or on an individual.

## Results

Broadwick was used to develop a generic compartmental model of disease spread through a network of animal holdings in Scotland (Soho), incorporating movements of individual cattle through the network. Soho was created to understand, through the use of the cattle movement network, how the disease transmission patterns change from a slow spreading to a fast spreading disease with different locations for the initial outbreak sources. It uses an SIR epidemiological model where it considers three compartments for the number of susceptible (S), infectious (I) and recovered (R) individuals. It uses movement data from the Cattle Tracing System database for Scotland (provided by RADAR [22]), which consists of 1.38 million animals at the beginning of 2005 and each simulation incorporates more than 2000 cattle movements per day. The duration of each simulation run can vary between 1 to 12 hours (depending if it is simulating a fast or a slow disease, respectively). More details about the Soho model can be found in (Salvador et al. In Prep.)

## Conclusions

Broadwick offers a computational framework that allows researchers to develop complex mathematical and simulation models of disease spread without having to worry about technical coding issues such as trying to efficiently read large datasets or develop a stochastic algorithm thus freeing time to devote to the model itself. Broadwick contains many packages that can be used as required it is designed to be flexible, not putting any requirement on how the user uses it, e.g. it contains it’s own entry point and configuration and data handling routines but these can be ignored and Broadwick used as a library of useful packages. It is a framework that provides considerable flexibility in developing epidemiological models, either for animal or human epidemiology. Broadwick continues to be actively developed with distributed computing abilities planned for future versions.

## Availability of data and materials

The Broadwick source and accompanying examples are freely available at http://epicscotland.github.io/broadwick.html under the Apache License, Version 2.0 license.

## References

[1] Bernoulli D, Blower S (2004). An attempt at a new analysis of the mortality caused by smallpox and of the advantages of inoculation to prevent it. Rev Med Virol.

[2] Datta S, Bull JC, Budge GE, Keeling MJ (2013). Modelling the spread of American foulbrood in honeybees. J R Soc Interface.

[3] Riley S (2007). Large-scale spatial-transmission models of infectious disease. Science.

[4] Kleczkowski A, Gilligan CA (2007). Parameter estimation and prediction for the course of a single epidemic outbreak of a plant disease. J R Soc Interface.

[5] Grenfell BT, Kleczkowski A, Gilligan CA, Bolker BM (1995). Spatial heterogeneity, nonlinear dynamics and chaos in infectious diseases. Stat Methods Med Res.

[6] Vespignani A, Colizza V, Barrat A, Barthe M (2005). The role of the airline transportation network in the prediction and predictability of global epidemics. Science.

[7] Ferguson NM, Donnelly CA, Anderson RA. The Foot-and-Mouth Epidemic in Great Britain: Pattern of Spread and Impact of Interventions. Science; 292(5519):1155–1160. doi:10.1126/science.1061020. 10.1126/science.1061020 11303090

[8] Ypma RJ, van Ballegooijen WM, Wallinga J (2003). Relating phylogenetic trees to transmission trees of infectious disease outbreaks. Genetics.

[9] Keeling MJ, Woolhouse MEJ, Shaw DJ, Matthews L, Chase-Topping M, Haydon DT, et al. Dynamics of the 2001 UK Foot and Mouth Epidemic: Stochastic Dispersal in a Heterogeneous Landscape. Science; 294(5543):813–817. doi:10.1126/science.1065973. 10.1126/science.1065973 11679661

[10] Colizza V, Barrat A, Barthélemy M, Vespignani A. The role of the airline transportation network in the prediction and predictability of global epidemics. PNAS; 103(7):2015–2020. doi:10.1073/pnas.0510525103. 10.1073/pnas.0510525103 PMC1413717 16461461

[11] Biek R, O’Hare A, Wright D, Mallon T, McCormick C, Orton RJ (2012). Whole Genome Sequencing Reveals Local Transmission Patterns of Mycobacterium bovis in Sympatric Cattle and Badger Populations. PLoS Pathogens.

[12] Morelli MJ, Thébaud G, Chadoeuf J, King DP, Haydon DT, Soubeyrand SA (2012). Bayesian Inference Framework to Reconstruct Transmission Trees Using Epidemiological and Genetic Data. PLoS Comput Biol.

[13] Bajardi P, Barrat A, Natale F, Savini L, Colizza V (2011). Dynamical Patterns of Cattle Trade Movements. PLoS ONE.

[14] Cauchemez S, Ferguson NM (2012). Methods to infer transmission risk factors in complex outbreak data. J R Soc Interface.

[15] Cottam EM, Thébaud G, Wadsworth J, Gloster J, Mansley L, Paton DJ (2008). Integrating genetic and epidemiological data to determine transmission pathways of foot-and-mouth disease virus. Proc Biol Sci.

[16] Apache Maven. http://maven.apache.org.

[17] Marjoram P, Molitor J, Plagnol V, Tavaré S (2003). Markov chain Monte Carlo without likelihoods. Proc Natl Acad Sci USA.

[18] Gamma E, Helm R, Johnson R, Vlissides J. Design patterns: elements of reusable object-oriented software: Addison-Wesley Longman Publishing Co. *ISBN:0-201-63361-2*.

[19] Cattle Tracing System. Defra. https://secure.services.defra.gov.uk/wps/portal/ctso.

[20] ScotEID - Scottish EID Livestock Traceability Research https://www.scoteid.com.

[21] One-JAR. http://one-jar.sourceforge.net.

[22] RADAR (Rapid Analysis and Detection of Animal-related Risks) is an information management system which has been developed to collect and collate veterinary surveillance data from a number of different sources around the UK.

